# Pleistocene Legacies in the Contemporary Population Genetic Structure of an Acid‐Specialised Crayfish, the Threatened Sand Yabby *Cherax robustus*

**DOI:** 10.1002/ece3.74364

**Published:** 2026-09-17

**Authors:** Grace Smith, Tomer Ventura, Catherine M. Yule

**Affiliations:** ^1^ Centre for Bioinnovation, School of Science Technology and Engineering University of the Sunshine Coast Sippy Downs Queensland Australia

**Keywords:** biogeography, conservation, crayfish, freshwater, population genetics

## Abstract

Following decades of clearing, fragmentation and degradation of coastal heathlands in southeast Queensland, many endemic species are now threatened with extinction, including the vulnerable sand yabby *Cherax robustus* (Parastacidae), a freshwater crayfish restricted to 200km^2^ of acidic wetlands. Dramatic fluctuations in the distribution and extent of these wetlands, particularly during the glacial cycles of the Pleistocene, likely shaped the distribution and genetic structure of acid‐specialist species, yet their historic and contemporary connectivity remains poorly understood, despite these species being particularly sensitive to habitat fragmentation and environmental change. Here, we investigate the genetic structure of two island and two mainland *C. robustus* populations within peatlands using DArTseq next‐generation DNA sequencing. Analysis of over 7000 SNP markers indicates population divergence occurred during the mid‐to‐late Pleistocene, with no detectable gene flow between most sites for between 50,000 and 120,000 years. Consistent with long‐term isolation, we found slight heterozygote deficiency in all populations, and evidence of genetic erosion in only one population, which should continue to be monitored, though the effect appears to predate European arrival in Australia. Evidently, sand yabbies are not naturally rare within stable environments but have become vulnerable through anthropogenic pressures of habitat loss and degradation. The strong population structuring observed means that each population represents a unique genetic lineage and should be managed as an independent unit. Protecting all remaining habitat and preventing any further degradation is therefore critical to the conservation of this species as well as the wider ecosystem.

## Graphical abstract

This study describes the contemporary genetic structure of a vulnerable short‐range endemic crayfish which is restricted to the heavily fragmented heathland‐peatland complexes of southeast Queensland across a range of approximately 200 km^2^. Using high‐resolution genome‐wide SNP sequencing, we estimate that population divergence in this species coincides with the mid‐to‐late Pleistocene sea level oscillations that fragmented the now‐offshore sand barrier islands. We demonstrate that populations are highly genetically diverged, showing minor signs of inbreeding, and find no evidence of contemporary gene flow between sites.

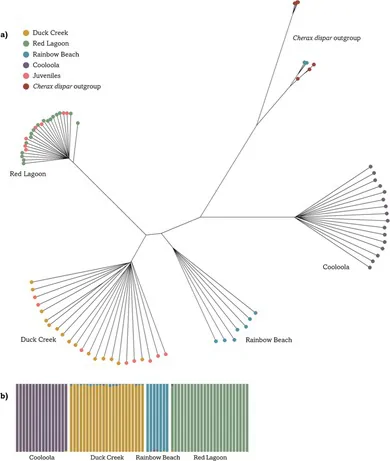

## 1 Introduction

### 1.1 A Brief History of the Coastal Sand Islands of Eastern Australia

With a dynamic geological and ecological history spanning more than 1.2 million years, the coastal sand barrier islands of Eastern Australia are a unique geological setting to study biogeography (Boyd et al. 2004; Ellerton et al. 2022; Shulmeister et al. 2024). These coastal dune systems are both the largest and oldest in the world, comprising K'gari (previously known as Fraser Island) and Cooloola in the north, and several large islands further south including Yarun (Bribie Island), Mulgumpin (Moreton Island) and Minjerribah (North and South Stradbroke Islands).

These sandmasses support a discontinuous mosaic of acidic, nutrient‐deficient ecosystems harbouring highly specialised flora and fauna, including disjunct pockets of highly acidic peatlands within more broadly distributed waterlogged heathlands (Coaldrake 1961; Pearl et al. 2024). Though now disjointed, these landscapes were often connected via paleodrainages during the Pleistocene when glacial eustatic oscillations saw the sea level periodically drop by around 120 m below present, exposing the continental shelf extending 40 km east of the modern coastline (Carter and Johnson 1986; Hanson et al. 2023; Lewis et al. 2013; Passos et al. 2019; Payenberg et al. 2006). As obligate freshwater lineages are constrained by physical processes and catchment boundaries, shifting biogeographic barriers and paleodrainage connectivity created by oscillating sea levels during the Pleistocene likely shaped contemporary species distributions before the transition to relatively stable Holocene conditions (Coaldrake 1961; Waters et al. 2026).

Most local peatlands initiated between 6000 and 12,000 years ago during this relatively stable Holocene climate, formed through organic matter deposition in river valleys and perched lake basins (Chalmers et al. 2025; Moss et al. 2015). Local peatland ecosystems were only formally recognised in 1996, by which point much of the original heathland extent had already been cleared or developed. Despite the ecological distinction between the two ecosystems, peatlands are often conflated with broader heathland communities, and are rarely recognised in their own right (Sattler 1999; UNEP 2022). The two ecosystems seem superficially analogous, and many aquatic and terrestrial species are distributed across both, reflecting shared environmental parameters such as chronic acidity, nutrient poverty, waterlogging and frequent fire. Peatlands are, however, fundamentally distinct, most significantly in their ability to sequester organic matter in peat layers at least 8 m deep, with recorded carbon density ranging between 300 and 3600 t per hectare, depending on humification, depth and age (Chalmers et al. 2025; Leach 2011; Tibby et al. 2017).

Since the 1960s, clearing for urban and agricultural expansion has locally destroyed nearly 40% of mainland heathland habitat (Bryan 1973; McGuire 1968; Pearl et al. 2022), though the proportion of this loss that may have contained peat substrates is challenging to quantify. With extremely specialised habitat requirements and low vagility, the species endemic to these remaining ‘island’ pockets of acidic wetlands are especially vulnerable to fragmentation‐induced genetic bottlenecks, inbreeding and localised extinctions (Frankham 1998; Renwick 2006). Nearly all locally endemic fish, frog and crayfish species have been listed as threatened under state and/or federal legislation, primarily due to decline in habitat quality, availability and connectivity (Knight et al. 2012; Marshall et al. 2011; Meyer 2006; Renwick 2006; Shuker et al. 2016). Despite the cultural and ecological significance of these ecosystems, there is a shortfall of information relating to their more recent biogeography and genetic trajectory, especially considering their proximity to major urban centres including Noosa, Brisbane and the Sunshine Coast.

### 1.2 Population Structuring Across Acid Islands

A progressively complete, precise and comprehensive understanding of genetic and ecological relationships is increasingly informing how conservation decisions are made (Avise 1995; Carrier et al. 2018; Feutry et al. 2020; Mijangos et al. 2015). The genetic structure of populations can be used to understand the factors driving distribution and inform the independent conservation of discrete populations, particularly when compounded with low dispersal capacity (Devloo‐Delva et al. 2023; Fast et al. 2023; Feutry et al. 2020; Oliveira et al. 2020). Ice age events are well documented to leave strong genetic traces in contemporary populations (Hewitt 2000); however, case studies are unsurprisingly fewer in the southern hemisphere. Pleistocene ice age events in Australia generally manifested as cycles of increasing aridity rather than continental glaciation, and genetic signatures in southern Australia consequently show biota persisted through climatic change via reliance on dynamic mosaic refugia (Byrne 2008), rather than the large‐scale range shifts and post‐glacial recolonisation typical of northern hemisphere biota (Hewitt 2000).

A strong spatial intraspecific divide has been described in freshwater macroinvertebrates and fish on both K'gari (north–south divide) and Minjerribah (east–west divide) (Marshall et al. 2011; Page et al. 2012), attributed to geographical barriers and a lack of hydrological connectivity, rather than overland distance. Freshwater fish, frogs, crayfish and shrimp show strong differentiation between disjunct wetlands, with limited gene flow even between geographically proximate sites (Dawkins et al. 2017; Garvie 1998; Hughes et al. 1999; Knight et al. 2012; Page and Hughes 2007a, 2007b; Renwick 2006).

Though sympatric species might expectedly show concordant phylogeographic patterns of simultaneous vicariance in response to landscape shifts (Waters et al. 2026), existing studies present somewhat conflicting patterns of Pleistocene connectivity and contemporary dispersal capacity. Molecular clock calculations generally place divergences during the Pleistocene, or for some species during the Pliocene (> 2.5 million years ago) and generally do not support recent connectivity during the Holocene (e.g., Page et al. 2004, Page et al. 2012; Renwick 2006; Sharma and Hughes 2011). Populations of many species, such as *Caridina* freshwater shrimp or the wallum froglet ( *Crinia tinnula* ) appear to have remained reproductively isolated throughout Pleistocene sea‐level oscillations, despite apparently continuous historical habitat during glacial low stands (Page and Hughes 2007a, 2007b; Renwick 2006). Anciently diverged swamp crayfish (*Tenuibranchiuris glypticus*) populations even comprise multiple clades and subclades across their range, which likely represent cryptic novel species and potentially a novel genus (Dawkins et al. 2017). By contrast, populations of Oxleyan Pygmy Perch ( *Nannoperca oxleyana* ) and certain *Caridina* sp. may have only become isolated as recently as the Pleistocene–Holocene boundary (Hughes et al. 1999; Woolschot et al. 1999). It is not uncommon for sympatric species to have vastly different life histories, and therefore different population structure in response to the same environmental changes, even in closely related taxa (e.g., Sharma and Hughes 2009). Contrasting patterns of connectivity in sympatric species may reflect differences in extrinsic ecological processes, such as historic distribution, or intrinsic physiological differences between species, such as dispersal capacity (Waters et al. 2026).

### 1.3 Case Study: The Sandy Yabby *Cherax robustus*

**FIGURE 1:**
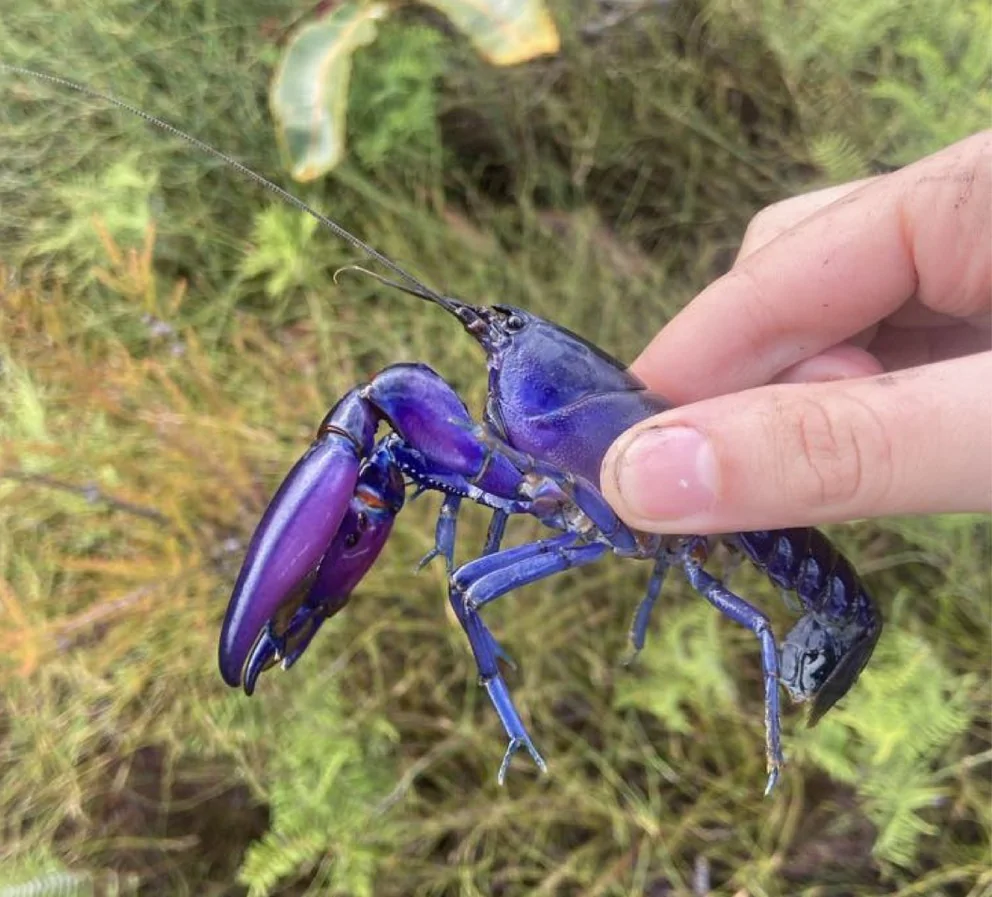
Large male sand yabby ( *Cherax robustus* ) sampled from Rainbow Beach for population genetic studies. Photograph by GS.

Here, we focus on the sand yabby *Cherax robustus* (Parastacidae), which is one of the most conspicuous species in the system (Figure 1), and a useful indicator of ecosystem health given its abundance and sympatry with other species of conservation concern (Garvie 1998). Despite occurring across an estimated area of only 212km^2^, *C. robustus* is relatively abundant within undisturbed habitat (Garvie 1998; Page 2021), and its burrows are significant landscape features, proposed as refugia during fire for sympatric aquatic vertebrates and invertebrates (Lowe et al. 2013; Page 2021). The long filamentous setae on the inner chelae imply a detritivorous diet, though this remains unconfirmed (Scully 2024, unpublished undergraduate thesis), and basic aspects of the crayfish's biology, including reproduction, growth rate and population sizes, are otherwise largely undocumented.

Despite apparent resilience within intact habitat, *C. robustus* was formally listed as vulnerable under both the Commonwealth EPBC Act and the Queensland Nature Conservation Act in 2023, making it the most recent acid‐specialist to receive threatened status, joining all four acid frogs and two fish listed previously. The primary cause of decline across this entire guild of species is range restriction, with ongoing threats including habitat loss, altered hydrological conditions and diminished habitat quality (e.g., Knight et al. 2012; Marshall et al. 2011; Meyer 2006; Renwick 2006; Shuker et al. 2016).

Two previous unpublished studies on *C. robustus* identified multiple discrete lineages with no haplotype sharing between populations (Garvie 1998; Bentley 2007), indicating long‐term isolation consistent with other acid‐specialists. Both studies identified maximum haplotype diversity on Bribie Island and two divergent lineages on K'gari, with a lack of haplotype sharing between populations indicating that populations had likely been isolated since at least the last glacial retreat (Page et al. 2012). These findings align with the predicted Death Valley model of potential connectivity (Hughes et al. 2013), as individuals are unlikely to disperse between sites due to lack of hydrological connectivity, resulting in strong differentiation by isolation.

As coastal development continues, it is important to understand how historical population dynamics influence contemporary genetic diversity. Conservation legislation typically treats threatened species as a single conservation unit, when it is likely that independent units would better represent conservation needs for disjunct freshwater lineages (i.e., Page et al. 2004). Tractability and abundance in the ecosystem make *C. robustus* considerably more amenable to genetic sampling than their vertebrate counterparts, particularly as pleopods can be collected non‐lethally and regenerate rapidly following moult. While previous genetic studies have relied on mitochondrial markers, advances in high‐throughput sequencing now enable low‐cost genome‐wide assessment of genetic diversity, even for non‐model organisms lacking reference genomes (Edet et al. 2018; Kilian et al. 2012). Single nucleotide polymorphism (SNP) datasets generated through genotyping‐by‐sequencing platforms provide orders of magnitude more markers than traditional approaches, allowing more precise estimates of population parameters including effective population size and divergence times (Devloo‐Delva et al. 2023; Reitzel et al. 2013). Here, we build on previous studies by using population genomic analysis of genome‐wide SNP markers to test whether genetic structure in *C. robustus* reflects Pleistocene sea‐level dynamics, estimate population divergence times and assess genetic health across four populations.

## 2 Methods

### 2.1 Sample Collection and Sequencing

**FIGURE 2:**
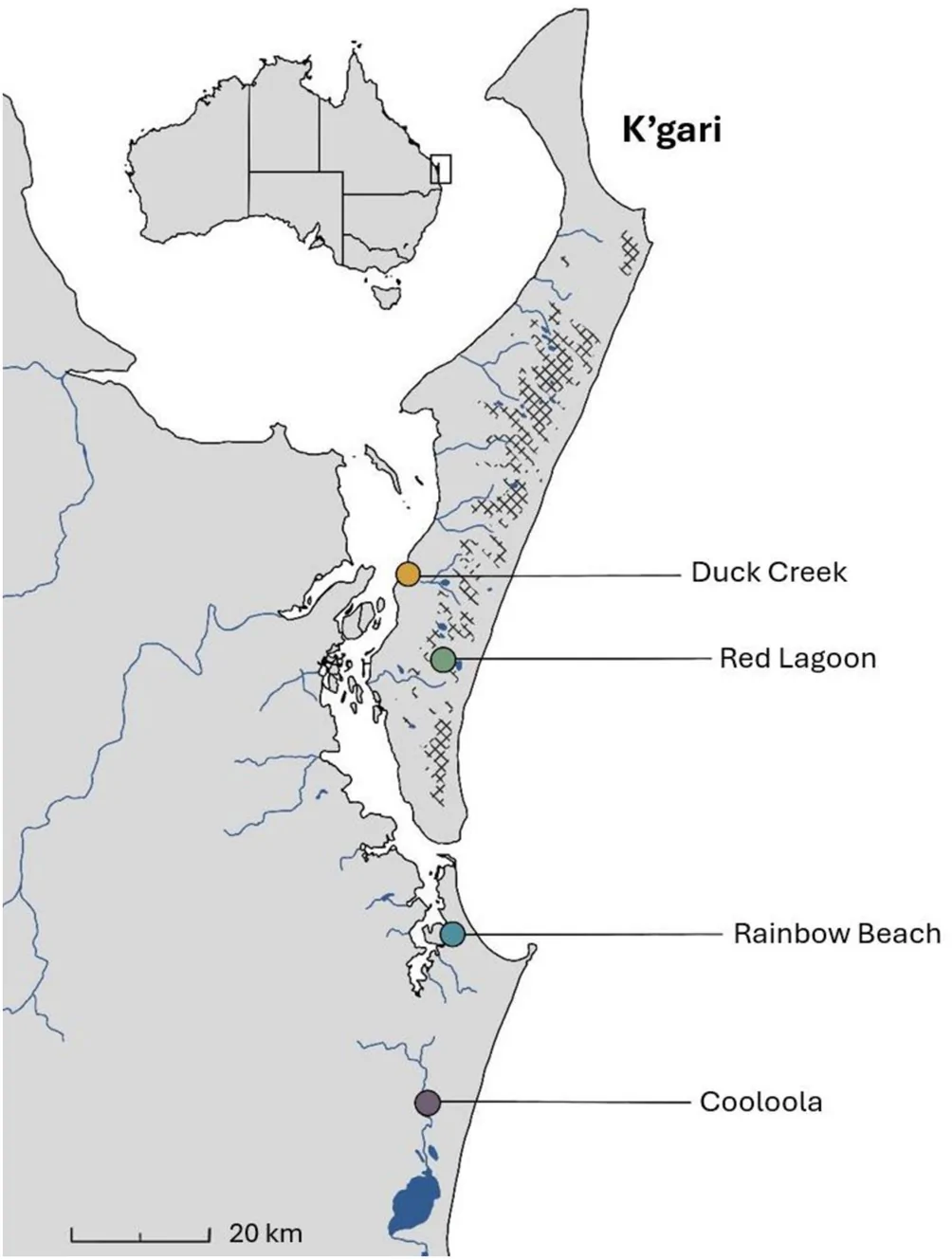
Crayfish were sampled from two peat sites on K'gari (Duck Creek (*n* = 15 adults, 8 juveniles), Red Lagoon (*n* = 17 adults, 7 juveniles)) and two on the mainland (Rainbow Beach (*n* = 7 adults), Cooloola (*n* = 16 adults)). Important creeks, lakes and drainages are indicated in blue. K'gari's central dune ridge (> 135 m) is indicated by black hatching.

Muscle tissue was collected from a total of 71 *Cherax robustus* across four sampling locations: two mainland (Cooloola, Rainbow Beach) and two K'gari peatlands (Red Lagoon, Duck Creek) in southeast Queensland, Australia (Figure 2; Table 1). Muscle tissue from an additional 10 *Cherax dispar* was collected from Rainbow Beach as an outgroup comparison. Collections within Great Sandy National Park were conducted under Queensland Parks and Wildlife Services Research Permit P‐PTUKI‐100757935.

**TABLE 1:** Genetic diversity metrics across *Cherax robustus* populations. *n* = sample size, He = expected heterozygosity (0–1), Ho = observed heterozygosity (0–1), F_IS_ = inbreeding coefficient (0–1), Polymorphic = number of polymorphic loci, % Poly = percentage of polymorphic loci, Allelic richness = mean alleles per locus (1–2).

	GPS	*n*	Ho	He	He%	F_IS_	F_IS_SD	Loci	Poly	Mono	% poly	*n*_alleles	Allelic richness	Private alleles
Duck Creek	−25.443, 153.011	23	0.155	0.186	83.3	0.174	0.32	7641	4271	3370	55.9	5044	1.50	21
Red Lagoon	25.555, 153.061	24	0.038	0.039	97.4	0.053	0.25	7641	1204	6437	15.8	2446	1.11	4
Rainbow Beach	−25.917, 153.074	7	0.167	0.192	87.0	0.170	0.40	7635	4284	3351	56.1	4856	1.66	79
Cooloola	−26.146, 153.051	16	0.122	0.151	80.8	0.195	0.35	7639	3467	4172	45.4	4668	1.41	1173

Crayfish were collected using unbaited collapsible box traps deployed for 45‐min intervals. For non‐lethal tissue sampling, pleopods (swimming legs) weighing 100‐500 mg were removed using sterilised straight‐edged scissors. Sex was determined by gonopore configuration (see Smith et al. 2023), and individuals were classified as adults (gonopores visible) or juveniles (gonopores not visible). Tissue samples were preserved in 90% ethanol and stored at room temperature until DNA extraction. All crayfish were immediately released after sampling.

Tissue samples (30–50 mg) were sent to Diversity Array Technology Pty Ltd. (Canberra, Australia) for genotyping (https://www.diversityarrays.com/papers_category/research/). Genomic DNA was extracted using a custom in‐house CTAB‐based extraction protocol. Sequencing was performed by DArTseq, combing DArT complexity reduction with high‐density next‐generation sequencing (2.5 million reads per sample) on the Illumina HiSeq2500 platform. Approximately 50,000 DNA fragments from DArT genomic representations were assayed for polymorphism. Raw fastq data returned for all samples are available as Data S1.

### 2.2 Data Analysis

Returned DArTseq sequences were further filtered and processed in R v4.5.1 (R Core Team, 2026) using *dartRverse* v1.0.6 (Gruber et al. 2018; Mijangos et al. 2022). All scripts are available via Zenodo under the DOI: 10.5281/zenodo.21576968. SNP loci were filtered using the following criteria: *monomorphic; secondaries; reproducibility* > *0.98; call rate* > *0.75; minor allele frequency* < *0.05* to create a dataset of all individuals from both species (referred to below as ‘all SNPs’) which contained 13,869 loci and 80 individuals, as a single *C. dispar* individual which returned zeros at all loci was removed. A data subset was filtered more stringently using an additional step, *individual call rate* > *0.75*, to create a dataset of *C. robustus* individuals only (referred to below as ‘*Cr SNPs*’), which contained 7641 loci and 70 individuals.

Using *all SNPs*, an unrooted neighbour‐joining tree was constructed based on pairwise Euclidean genetic distances using the neighbour‐joining algorithm (Saitou and Nei 1987) in *ape* v5.8–1 (Paradis and Schliep 2018).

The *Cr SNPs* data subset was used for the following analyses: Hardy–Weinberg equilibrium (HWE) was tested for each locus within each population using *pegas* v1.3 (Paradis 2010). Juveniles were excluded from HWE testing as they were collected from multiple sites and therefore showed artificially elevated ‘structuring’ consistent with the Wahlund effect (Garnier‐Géré and Chikhi 2013). Within the adult populations, all loci were retained for subsequent analyses as none showed significant deviation from HWE across three or more populations. Juveniles were pooled with adult populations for subsequent analyses. All loci were assumed to be autosomal as no statistically significant sex‐linked loci were detected under a ZW sex‐determination model.

Heterozygosity reports were generated across populations, calculating expected heterozygosity (He), observed heterozygosity (Ho), degree of inbreeding (F_IS_) and number of polymorphic loci. Rarefied allelic richness was calculated using the *hierfstat* package (Goudet 2005). Pairwise F_ST_ values between adult populations were calculated using 1000 bootstrap replicates (Weir and ockerham 1984). sNMF analysis using LEA (Frichot and François 2015) tested *K* = 1 to *K* = 6 with 10 repetitions per K value and cross‐entropy validation. *K* = 4 was identified as the optimal number of genetic clusters, corresponding to the four sampling locations. Individual ancestry Q‐matrices were visualised using sNMF admixture bar plots.

Pairwise F_ST_ genetic distances were calculated between all population pairs. To estimate time since divergence, pairwise coalescent‐based analyses were conducted using the fastsimcoal2 coalescent simulator (Excoffier et al. 2021) accessed through the strataG R package (Archer et al. 2017). Prior to analysis, loci with any missing data were removed, reducing the dataset from 7641 to 3418 loci.

For each population pair, a joint minor allele frequency site frequency spectrum (SFS) was computed from the filtered genotype data. A simple two‐population isolation model was specified with no migration and no population growth, consistent with a vicariance scenario in which populations were separated by geographical barriers with no subsequent gene flow. A mutation rate of 2.5e‐8 per site per generation was used, as widely applied when using the fastsimcoal2 simulator with SNP data (e.g., García‐Ulloa et al. 2021; Josić et al. 2024; Nunziata and Weisrock 2018; Wang et al. 2025).

For each pair, the estimation was run 10 independent times using 100,000 simulations per run, retaining the highest maximum log‐likelihood as the best estimate. Divergence times in generations were converted to years by multiplying by an estimated generation time of 4 years, based on observations in other non‐invasive *Cherax* species (Beatty et al. 2005; Duffy et al. 2025; Morgan and Beatty 2005). Effective population sizes were independently estimated using linkage disequilibrium (gl.LDNe) in dartR.

## 3 Results

### 3.1 Diversity Metrics

**FIGURE 3:**
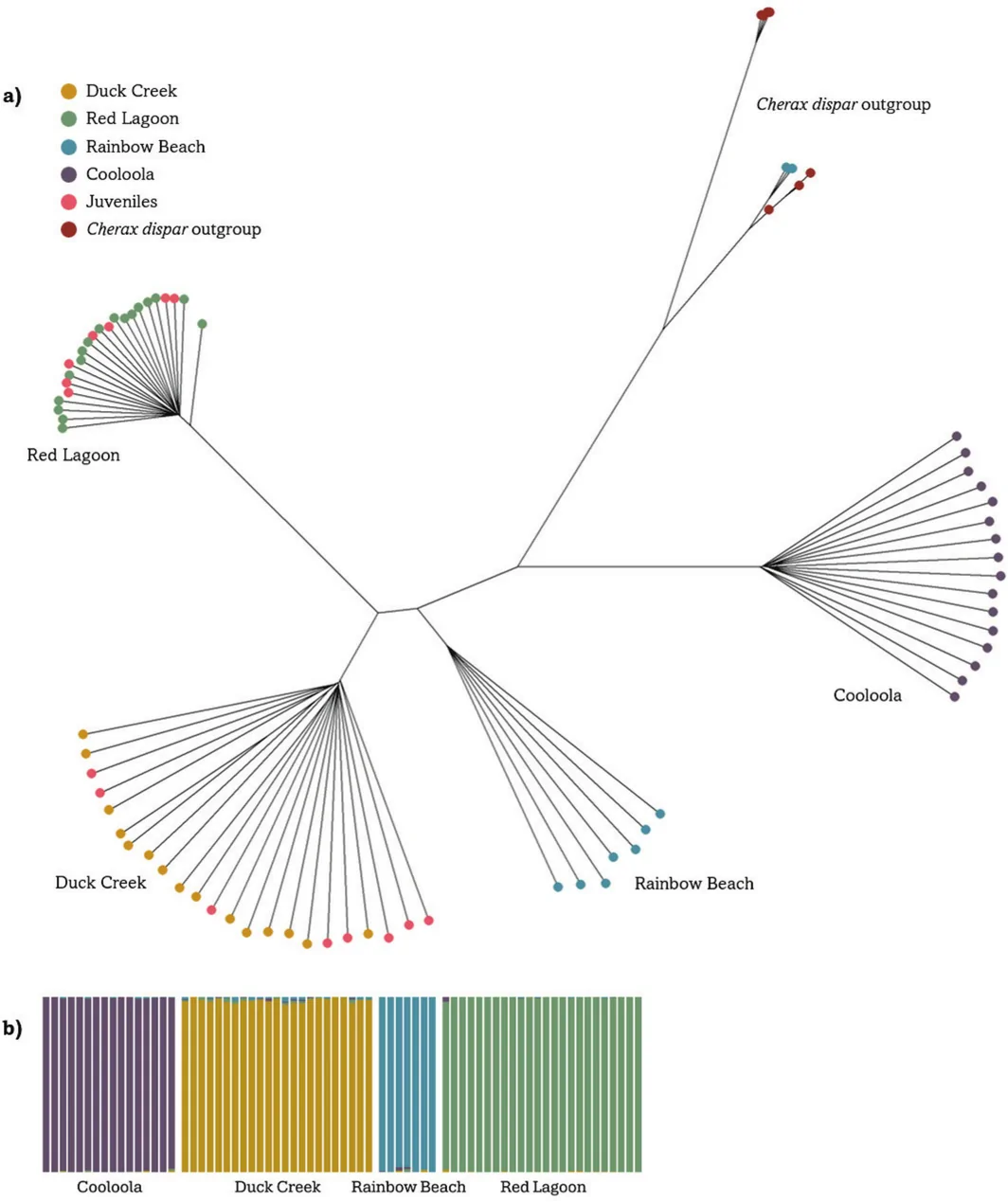
Population genetic structure of *Cherax robustus* . (a) Unrooted neighbour‐joining tree based on Euclidean genetic distances between individuals. Each dot represents an individual, and branches are proportional to genetic distance. Each population forms a discrete clade, and juveniles (pink) cluster with local adults. The *C. dispar* outgroup (red) forms a separate clade with two internal lineages. (b) Individual ancestry proportions from sNMF analysis (*K* = 4). Each vertical bar represents one individual, grouped by population. Colours indicate ancestry from four inferred clusters. 80 crayfish were sampled in total: Cooloola (*n* = 16 adults), Duck Creek (*n* = 15 adults, 8 juveniles), Rainbow Beach (*n* = 7 adults), Red Lagoon (*n* = 17 adults, 7 juveniles), *Cherax dispar* outgroup (*n* = 10 adults).

A total of 80 crayfish were analysed from four peat sites (Cooloola (*n* = 16 adults), Duck Creek (*n* = 15 adults, 8 juveniles), Rainbow Beach (*n* = 7 adults) and Red Lagoon (*n* = 17 adults, 7 juveniles)) plus 10 *C. dispar* individuals as an outgroup. Three individuals collected from Rainbow Beach and initially identified as *C. robustus* in the field clustered genetically with the *C. dispar* outgroup (Figure 3), indicating field misidentification due to morphological similarity, particularly for small individuals (< 30 mm OCL). After filtering, 13,869 loci were retained across both species, and 7641 loci excluding the *C. dispar* outgroup. Mean read depth was 32.9 and mean missing data was 3.5% per individual.

Genetic diversity metrics across populations are reported in Table 1. Observed heterozygosity (Ho) ranged from 0.038 (Red Lagoon) to 0.167 (Rainbow Beach) and was consistently slightly lower (within > 80%) than expected heterozygosity (He), ranging from 0.039 (Red Lagoon) to 0.192 (Rainbow Beach). All populations showed a slight heterozygote deficiency (F_IS_ = 0.053–0.195) consistent with long‐term isolation, mild inbreeding, or population substructure.

Despite having the smallest sample size (*n* = 7), Rainbow Beach had the most polymorphic loci (4284; 56.1%) and highest rarefied allelic richness (1.66 alleles per locus). In contrast, Red Lagoon showed depleted allelic richness (1.11 alleles per locus) and only 1204 polymorphic loci (15.8%) despite having the largest sample size. Red Lagoon also had the fewest private alleles (4) whereas Duck Creek and Rainbow Beach had moderate numbers (21 and 79 respectively). Cooloola had a far greater number of private alleles (1173), consistent with long‐term isolation or limited gene flow.

### 3.2 Population Structure

A neighbour‐joining tree based on Euclidean genetic distances revealed four discrete clades corresponding to the four sampling localities (Figure 3a). Each population formed a distinct cluster with no overlap of individuals from different sites. Individuals at Red Lagoon showed the shortest internal branch distances between individuals (proportional to genetic distance) consistent with observed lowered genetic diversity metrics. Juveniles clustered exclusively with adults from their respective collection sites, indicating no contemporary dispersal among populations. The *C. dispar* outgroup formed a separate clade with two internal lineages, reflecting collection localities. Genetic distance to *C. robustus* populations was comparable to interpopulation distances within *C. robustus* , reflecting extreme differentiation between study populations.

Population structure analysis using sNMF identified *K* = 4 as the optimal number of genetic clusters (Figure 3b). Each sampling locality corresponded to a single genetic cluster with minimal admixture. Individual ancestry assignments showed strong population structure with negligible contemporary gene flow among sites. Where individuals showed slight mixed ancestry, it was primarily between Duck Creek and Rainbow Beach, the two populations with lowest pairwise genetic differentiation (F_ST_ = 0.30) (Table 2).

**TABLE 2:** Pairwise F_ST_ values among four *Cherax robustus* populations. All comparisons were highly significant (*p* < 0.001 based on 1000 bootstrap replicates).

	Duck Creek	Red Lagoon	Rainbow Beach	Cooloola
Duck Creek		0.58	0.30	0.63
Red Lagoon	0.58		0.67	0.81
Rainbow Beach	0.30	0.67		0.54
Cooloola	0.63	0.81	0.54	

### 3.3 Genetic Distance

Pairwise F_ST_ values ranged from 0.30 (Duck Creek‐Rainbow Beach) to 0.81 (Red Lagoon‐Cooloola), indicating strong genetic differentiation between all population pairs (Table 2). All pairwise comparisons exceeded the widely accepted threshold of F_ST_ > 0.25 for very high differentiation (Wright 1965), with all except one pair exceeding 0.50. Duck Creek and Rainbow Beach showed the lowest differentiation (F_ST_ = 0.30), which could indicate more recent divergence or historical gene flow. Red Lagoon showed the highest divergence from all other populations (F_ST_ = 0.58–0.81) despite its central geographic location.

**FIGURE 4:**
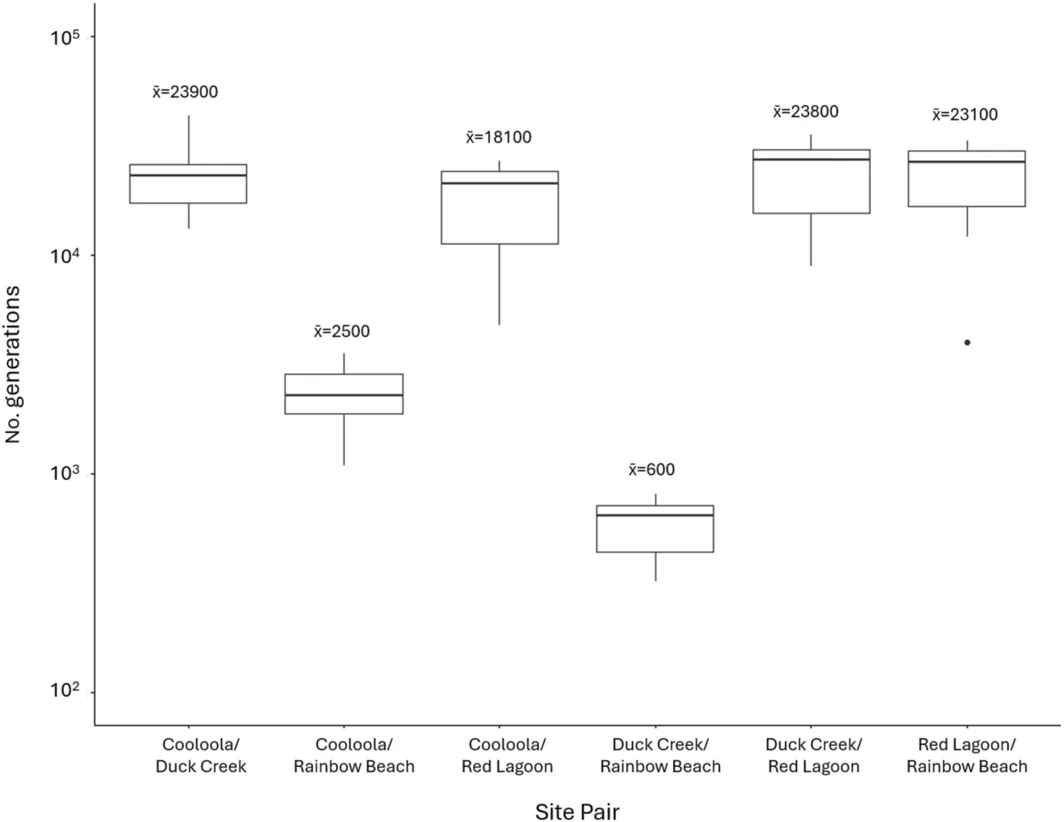
Generations since divergence in *Cherax robustus* population pairs estimated from 10 independent coalescent simulation runs per pair. Mean divergence time (x̄) for each pair is labelled to the nearest hundred.

**FIGURE 5:**
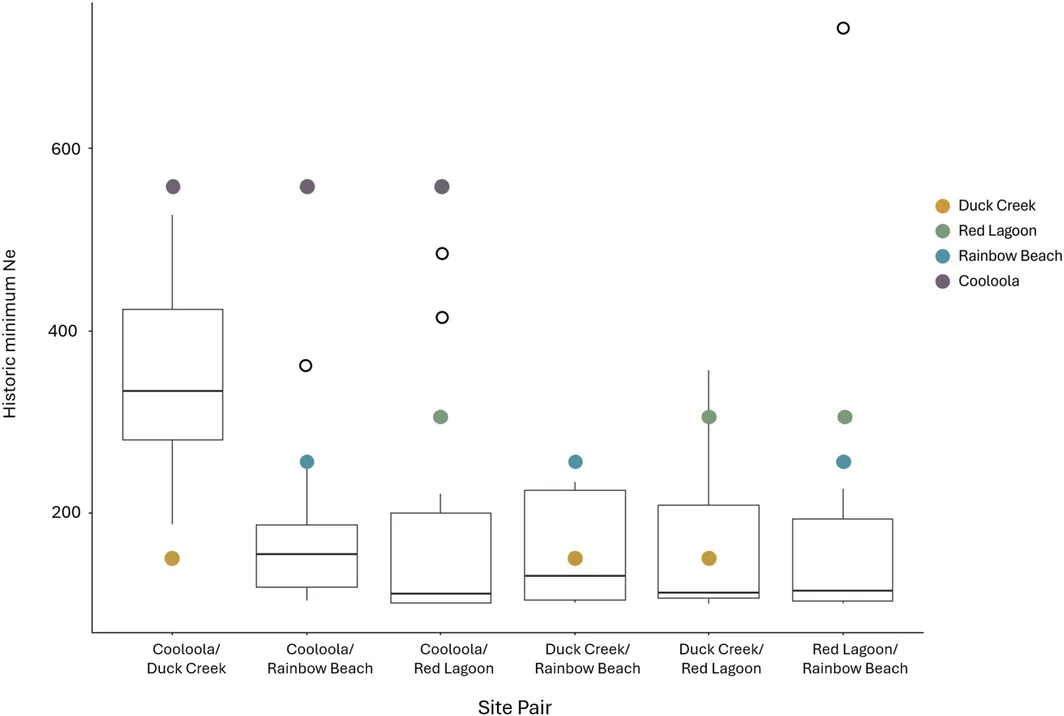
Historic minimum effective population size (Ne) estimated by coalescent simulation (boxplots) and by linkage disequilibrium (points, coloured by population). White circles indicate boxplot outliers. Both methods consistently estimate historic minimum Ne between 100 and 600 individuals across sites, with Cooloola having the largest historic Ne relative to the other populations.

Divergence time estimates based on coalescent simulations placed divergence between most population pairs within the window of 12,000 to 30,000 generations ago (Figure 4), equivalent to approximately 48,000–120,000 years assuming an average generation time of four years. These estimates consistently place population divergence during the mid‐to‐late Pleistocene. Consistent with the lowest pairwise F_ST_ estimates, the Rainbow Beach population showed a more recent divergence from both Cooloola (x̄ ≈2500 generations; ~10,000 years BP) and Duck Creek (x̄ ≈600 generations; ~2400 years BP). The divergence between the Rainbow Beach and Cooloola populations aligns with the Holocene–Pleistocene boundary, while the divergence between the Rainbow Beach and Duck Creek populations falls within the mid‐Holocene. Substantial uncertainty exists in these estimates given the assumptions inherent to the isolation model and the absence of a species‐specific mutation rate calibration. As an internal validity test, effective population size was estimated independently using both the coalescent simulations and the linkage disequilibrium method. Both methods estimated broadly consistent historic minimum effective population sizes within the window of 100 and 600 individuals across sites (Figure 5).

## 4 Discussion

### 4.1 Contemporary Genetic Structure of *Cherax robustus*

Genome‐wide SNP analysis found strong genetic structuring among *C. robustus* populations, with pairwise F_ST_ values ranging from 0.30 to 0.81, far exceeding the widely accepted threshold of 0.25 for very high divergence (Wright 1965). Neighbour‐joining analysis identified four discrete genetic clades corresponding to sampling localities, with no evidence of contemporary admixture between sites. This strong population structuring is consistent with earlier mitochondrial studies that reported no shared haplotypes among populations as well as multiple divergent lineages on K'gari (Garvie 1998; Bentley 2007).

Coalescent modelling estimates that population divergence for most study sites occurred between approximately 48,000 and 120,000 years BP during the mid‐to‐late Pleistocene, splitting prior to the Last Glacial Maximum which occurred between around 30,000 and 22,000 years BP (Passos et al. 2019). The Rainbow Beach population appears to be more recently diverged, being separated from Cooloola approximately 10,000 years BP, around the Pleistocene–Holocene boundary, and from Duck Creek approximately 2400 years BP. There is large uncertainty in these estimates due to the assumptions made for mutation rate, generation time and coalescent modelling. However, even conservative estimates place population divergence thousands of years prior to European settlement, indicating that contemporary genetic structure in the sampled populations reflects long‐term landscape processes rather than recent anthropogenic fragmentation.

Discrete sub‐populations were observed between all sampling localities (F_ST_ > 0.30), indicating long‐term isolation and independent evolutionary trajectories. Similar patterns of discrete genetic clades in the sympatric freshwater crayfish *T. glypticus* have been interpreted as evidence of cryptic diversity (Dawkins et al. 2017), however, subspecies delineation remains subjective and contentious (e.g., Frankham et al. 2012; Haig et al. 2006; Phillimore and Owens 2006). As no consistent morphological differentiation was detected among populations, the high degree of genetic divergence observed is likely a response to extrinsic landscape pressures, rather than intrinsic factors, as described in other local crustaceans (see Sharma and Hughes 2009). We therefore interpret these populations as long‐isolated subpopulations within a single species, shaped by landscape‐driven isolation rather than speciation.

Regardless of taxonomic interpretation, the magnitude of genetic differentiation and the absence of detectable gene flow indicate that each population represents a unique evolutionary lineage. Moritz (1994) defines evolutionarily significant units (ESUs) as requiring both reciprocal monophyly in mitochondrial alleles and significantly divergent nuclear allele frequencies. Previous studies have identified discrete mitochondrial lineages between populations (Garvie 1998; Bentley 2007), which in tandem with the nuclear differentiation described here, satisfy the criteria for designation as ESUs. Together, these complementary studies demonstrate that each population should be treated as an independent management unit, as a better reflection of their genetic diversity (Moritz 1994). This outcome is consistent with findings from the sympatric ornate rainbowfish (*Rhadinocentrus ornartus*), where isolated lineages were similarly recommended for management as independent ESUs (Page et al. 2004). In both cases, natural genetic rescue (introduction of new genetic material) among populations is extremely unlikely, and from a conservation perspective, management strategies should approach each population independently, rather than as a single unit.

### 4.2 Genetic Health in Isolated Populations

Despite prolonged isolation, all *C. robustus* populations retained at least moderate genetic diversity, with observed heterozygosity at least 80% of expected heterozygosity across all sites. A slight heterozygote deficiency was detected in all populations (F_IS_ = 0.053–0.195). Though F_IS_ estimates above 0.125 would indicate moderate (second order) inbreeding in vertebrates (Nichols 2017), scores regularly exceed 0.3 in freshwater crayfish (e.g., Bertocchi et al. 2008; Lovrenčić et al. 2022) and other invertebrates (Addison and Hart 2005; Olsen et al. 2020). Therefore, the observed heterozygote deficiency likely reflects extended isolation and possible sub‐structuring rather than concerning inbreeding depression or bottlenecking.

Red Lagoon showed reduced genetic diversity relative to other populations, including a lower proportion of polymorphic loci (15.8% compared to approximately 50% elsewhere) and the fewest private alleles. However, this population also showed the lowest heterozygote deficiency (F_IS_ = 0.05, compared with ~0.18 in other populations), so reduced allelic richness is unlikely to reflect recent inbreeding. Instead, this pattern could reflect prolonged isolation and historical genetic drift following early divergence from other populations. Geographically, Red Lagoon is positioned on the eastern side of K'gari's central dune ridge, where there is less available wetland habitat and little hydrological connectivity. Marshall et al. (2011) and Page et al. (2012) both observed similar divides associated with drainage boundaries and landscape features such as the central dune ridge.

The apparent tolerance of *C. robustus* populations to moderate genetic erosion should not be extrapolated to other acid‐specialists. Where population sizes are smaller, as may be the case for some co‐occurring freshwater fishes and amphibians, vulnerability to fragmentation‐induced genetic erosion is expected to be higher (Knight et al. 2012; Meyer 2006; Shuker et al. 2016). Low genetic diversity can reduce adaptive potential and increase vulnerability to inbreeding depression, environmental stochasticity and disease (Bijlsma and Loeschcke 2012; Frankham 1998), particularly under accelerating climate change pressures on coastal wetlands (IPCC AR6, 2025). While the reduced allelic diversity at Red Lagoon might predate anthropogenic fragmentation, future habitat degradation could exacerbate existing vulnerabilities, and the relative genetic stability observed in crayfish populations does not diminish the broader conservation urgency for peatland ecosystems.

### 4.3 Paleobiogeography

Estimates of population divergences place the splitting of most site pairs during the mid‐to‐late Pleistocene, broadly between ~48,000 and ~120,000 years BP. The Rainbow Beach population is apparently more recently diverged from Cooloola, approximately 10,000 years BP, coinciding with sea‐level stabilisation after the Last Glacial Maximum, as well as Duck Creek, around 2400 years BP. However, substantial uncertainty exists in these estimates due to the reliance on mutation rates extrapolated from other studies, assumptions in modelling simulations and average generation time estimated based on other *Cherax* species (Beatty et al. 2005; Duffy et al. 2025; Morgan and Beatty 2005). If *C. robustus* has slower molecular evolution or longer generation times than assumed, true divergence times may be older than estimated. Nevertheless, even conservative estimates indicate that populations maintained connectivity during much of the periodic low sea‐level stands that exposed continental shelf during the Pleistocene. Similar patterns of connectivity corridors are observed in other local taxa (Conroy et al. 2021; Hughes et al. 1999; Woolschot et al. 1999), though many co‐occuring taxa appear to have remained isolated for much or all of the Pleistocene (Dawkins et al. 2017; Page and Hughes 2007a, 2007b; Renwick 2006).

The least diverged pair in the present study were Duck Creek and Rainbow Beach, estimated at 600 generations or approximately 2400 years BP, which is notable given their separation by more than 50 km overland as well as the Great Sandy Strait, which has existed for at least 6000 years (Bryant 1992; Shulmeister et al. 2024), apparently predating the split between these populations. Possibly, divergence times here are underestimated due to uncertainty in coalescent modelling parameters and assumptions, and true divergence may have occurred closer to the formation of the strait. As an alternative hypothesis, connectivity may have persisted across the shallow western margin of K'gari during mid‐Holocene cyclonic La Niña conditions, when increased fluvial sedimentation from the Mary River may have facilitated connectivity via land bridges or shifting sandbars before the transition to more arid conditions around 3000 years BP (Barr et al. 2019; Brooke et al. 2008; Leonard and Nott 2016). Alternatively, assisted migration by the Butchulla people, who have lived on K'gari for at least 5000 years, is also a possibility, particularly if these crayfish were a food resource and were translocated between sites as a consequence.

Collectively, the contemporary genetic structure in *C. robustus* reflects long‐term responses to extrinsic processes such as sea‐level change, dune formation and drainage reorganisation during the Pleistocene, and possibly into the Holocene. Topographic features, such as K'gari's central dune ridge, and hydrological barriers, such as drainage boundaries and marine straits, appear to exert a stronger influence on genetic structure than overland distance, consistent with previous studies of local freshwater taxa (e.g., Marshall et al. 2011; Page et al. 2012) and the predicted Death Valley model of connectivity, in which individuals are unable to disperse between sites due to hydrological isolation (Hughes et al. 2013).

## 5 Conclusion

Based on the outcomes of previous mitochondrial assessments (Garvie 1998; Bentley 2007) and the nuclear assessment presented in the current study, we argue that each population represents an independent evolutionary lineage requiring separate management. Permanent hydrological barriers and the lack of evidence for contemporary gene flow indicate recolonisation following local extinction is extremely unlikely. The loss of any population therefore results in permanent extinction of unique genetic diversity.

We find slight heterozygote deficiency in all populations, indicating minor inbreeding consistent with prolonged isolation. While the observed genetic erosion appears to be caused naturally, the apparent resilience of *C. robustus* to prolonged isolation should not be extrapolated to other fauna with different life histories. Sympatric vertebrates with smaller population sizes and lower acceptable inbreeding coefficients (e.g., Nichols 2017 vs. Addison and Hart 2005) might be less resilient to extended isolation. These species should be independently assessed, though it is apparent that habitat protection is the most important factor for protecting these populations.

Although the observed population structuring appears to reflect natural processes, long‐term genetic monitoring should be considered to detect demographic decline or inbreeding progression. Further anthropogenic pressures could exacerbate existing vulnerabilities, reduce population sizes and increase demographic stochasticity. Climate change threatens further habitat degradation through altered hydrology, increased fire frequency and sea‐level rise, compounding risks for populations unable to disperse to suitable habitat, especially when there is so little available. Preventing further habitat clearing and degradation is therefore critical, as the loss of individual wetlands results in irreversible loss of genetic diversity and adaptive potential.

## Author Contributions

**Grace Smith:** formal analysis (lead), methodology (equal), writing – original draft (lead), writing – review and editing (lead). **Tomer Ventura:** funding acquisition (supporting), methodology (equal), supervision (equal), writing – review and editing (equal). **Catherine M. Yule:** funding acquisition (lead), methodology (equal), supervision (equal), writing – review and editing (equal).

## Funding

This work was supported by Queensland Government, CSAT22032.

## Conflicts of Interest

The authors declare no conflicts of interest.

## Supporting information


**Data S1:** Fastsimcoal outputs.

**Data S2:** SNP.

## Data Availability

Raw SNP data are supplied as Data S1, ‘SNP.csv’. Coalescent simulation outputs are supplied as Data S2, ‘fastsimcoal outputs.xlsx’. All R scripts used for analyses are available on Zenodo under the DOI: 10.5281/zenodo.21576968.

## References

[ece374364-bib-0001] Addison, J. A. , and M. W. Hart . 2005. “Spawning, Copulation and Inbreeding Coefficients in Marine Invertebrates.” Biology Letters 1, no. 4: 450–453. 10.1098/rsbl.2005.0353 PMC1626360 17148230

[ece374364-bib-0002] Archer, F. I. , P. E. Adams , and B. B. Schneiders . 2017. “Stratag: An r Package for Manipulating, Summarizing and Analysing Population Genetic Data.” Molecular Ecology Resources 17, no. 1: 5–11. 10.1111/1755-0998.12559 27327208

[ece374364-bib-0003] Avise, J. C. 1995. “Mitochondrial DNA Polymorphism and a Connection Between Genetics and Demography of Relevance to Conservation.” Conservation Biology 9, no. 3: 686–690.

[ece374364-bib-0004] Barr, C. , J. Tibby , M. J. Leng , et al. 2019. “Holocene El Niño–Southern Oscillation Variability Reflected in Subtropical Australian Precipitation.” Scientific Reports 9, no. 1: 1627. 10.1038/s41598-019-38626-3 PMC6367503 30733569

[ece374364-bib-0005] Beatty, S. J. , D. L. Morgan , and H. S. Gill . 2005. “Life History and Reproductive Biology of the Gilgie, *Cherax quinquecarinatus* , a Freshwater Crayfish Endemic to Southwestern Australia.” Journal of Crustacean Biology 25, no. 2: 251–262.

[ece374364-bib-0006] Bentley, A. I. 2007. “Phylogeographic Structure of Freshwater Crayfish of the Genus *Cherax* (Decapoda: Parastacidae) on the Mainland and Islands of Southeast Queensland. Honours thesis. Griffith School of Environment, Griffith University.”

[ece374364-bib-0007] Bertocchi, S. , S. Brusconi , F. Gherardi , F. Grandjean , and C. Souty Grosset . 2008. “Genetic Variability in the Threatened Crayfish *Austropotamobius italicus*: Implications for Its Management.” Fundamental and Applied Limnology 17: 153–164.

[ece374364-bib-0008] Bijlsma, R. , and V. Loeschcke . 2012. “Genetic erosion Impedes Adaptive Responses to Stressful Environments.” Evolutionary Applications 5, no. 2: 117–129. 10.1111/j.1752-4571.2011.00214.x PMC3353342 25568035

[ece374364-bib-0009] Boyd, R. , K. Ruming , S. Davies , T. Payenberg , and S. Lang . 2004. “Fraser Island and Hervey Bay‐a Classic Modern Sedimentary Environment.” Petroleum Exploration Society of Australia (PESA) Eastern Australasian Basins Symposium II. Adelaide.

[ece374364-bib-0010] Brooke, B. , D. Ryan , T. Pietsch , et al. 2008. “Influence of Climate Fluctuations and Changes in Catchment Land Use on Late Holocene and Modern Beach‐Ridge Sedimentation on a Tropical Macrotidal Coast: Keppel Bay, Queensland, Australia.” Marine Geology 251, no. 3: 195–208.

[ece374364-bib-0011] Bryan, W. 1973. “A Review of Research Findings Concerned With Pastoral Development on the Wallum of South‐Eastern Queensland.” Tropical Grasslands 7, no. 2: 175–194.

[ece374364-bib-0012] Bryant, E. 1992. “Last Interglacial and Holocene Trends in Sea‐Level Maxima Around Australia: Implications for Modern Rates.” Marine Geology 108, no. 2: 209–217.

[ece374364-bib-0013] Byrne, M. 2008. “Evidence for Multiple Refugia at Different Time Scales During Pleistocene Climatic Oscillations in Southern Australia Inferred From Phylogeography.” Quaternary Science Reviews 27, no. 27: 2576–2585.

[ece374364-bib-0014] Carrier, J. C. , M. R. Heithaus , and C. A. Simpfendorfer . 2018. Shark Research: Emerging Technologies and Applications for the Field and Laboratory. CRC Press.

[ece374364-bib-0015] Carter, R. , and D. Johnson . 1986. “Sea‐Level Controls on the Post‐Glacial Development of the Great Barrier Reef, Queensland.” Marine Geology 71, no. 1–2: 137–164.

[ece374364-bib-0016] Chalmers, G. , Z. Ghasemzadeh , D. Chittleborough , et al. 2025. “Carbon Stock, Subsurface Characteristics and Accommodation Settings of Sub‐Tropical Peatland Histosols, K'gari, Queensland Australia.” Geodermal Regional 40: e00913.

[ece374364-bib-0017] Coaldrake, J. E. 1961. The Ecosystem of the Coastal Lowlands (Wallum) of Southern Queensland. CSIRO.

[ece374364-bib-0018] Conroy, G. C. , R. W. Lamont , L. Bridges , D. Stephens , A. Wardell‐Johnson , and S. M. Ogbourne . 2021. “Conservation Concerns Associated With Low Genetic Diversity for K'gari–Fraser Island Dingoes.” Scientific Reports 11, no. 1: 9503. 10.1038/s41598-021-89056-z PMC8097078 33947920

[ece374364-bib-0019] Dawkins, K. L. , J. M. Furse , C. H. Wild , and J. M. Hughes . 2017. “A Novel Genus and Cryptic Species Harboured Within the Monotypic Freshwater Crayfish Genus Tenuibranchiurus Riek, 1951 (Decapoda: Parastacidae).” PeerJ 5: e3310. 10.7717/peerj.3310 PMC5445942 28560095

[ece374364-bib-0020] Devloo‐Delva, F. , C. P. Burridge , P. M. Kyne , et al. 2023. “From Rivers to Ocean Basins: The Role of Ocean Barriers and Philopatry in the Genetic Structuring of a Cosmopolitan Coastal Predator.” Ecology and Evolution 13, no. 2: e9837. 10.1002/ece3.9837 PMC9944188 36844667

[ece374364-bib-0021] Duffy, R. , B. Hodgson , A. Quinn , and M. Newman . 2025. “Successful Artificial Incubation and Juvenile‐Rearing of Dropped Eggs of a Critically Endangered Freshwater Crayfish ( *Cherax tenuimanus* ).” Marine and Freshwater Research 76, no. 12: MF25063.

[ece374364-bib-0022] Edet, O. U. , Y. S. A. Gorafi , S. Nasuda , and H. Tsujimoto . 2018. “DArTseq‐Based Analysis of Genomic Relationships Among Species of Tribe Triticeae.” Scientific Reports 8, no. 1: 16397. 10.1038/s41598-018-34811-y PMC6219600 30401925

[ece374364-bib-0023] Ellerton, D. , T. M. Rittenour , J. Shulmeister , et al. 2022. “Fraser Island (K'gari) and Initiation of the Great Barrier Reef Linked by Middle Pleistocene Sea‐Level Change.” Nature Geoscience 15, no. 12: 1017–1026.

[ece374364-bib-0024] Excoffier, L. , N. Marchi , D. A. Marques , R. Matthey‐Doret , A. Gouy , and V. C. Sousa . 2021. “fastsimcoal2: Demographic Inference Under Complex Evolutionary Scenarios.” Bioinformatics 37, no. 24: 4882–4885. 10.1093/bioinformatics/btab468 PMC8665742 34164653

[ece374364-bib-0025] Fast, K. M. , B. L. Fluker , B. R. Kuhajda , et al. 2023. “Conservation Genomics of the Threatened Trispot Darter ( *Etheostoma trisella* ).” Conservation Genetics 25: 291.

[ece374364-bib-0026] Feutry, P. , F. Devloo‐Delva , A. Tran Lu , et al. 2020. “One Panel to Rule Them All: DArTcap Genotyping for Population Structure, Historical Demography, and Kinship Analyses, and Its Application to a Threatened Shark.” Molecular Ecology Resources 20, no. 6: 1470–1485. 10.1111/1755-0998.13204 32492756

[ece374364-bib-0027] Frankham, R. 1998. “Inbreeding and Extinction: Island Populations.” Conservation Biology 12, no. 3: 665–675.

[ece374364-bib-0028] Frankham, R. , J. D. Ballou , M. R. Dudash , et al. 2012. “Implications of Different Species Concepts for Conserving Biodiversity.” Biological Conservation 153: 25–31.

[ece374364-bib-0029] Frichot, E. , and O. François . 2015. “LEA: An R Package for Landscape and Ecological Association Studies.” Methods in Ecology and Evolution 6, no. 8: 925–929.

[ece374364-bib-0030] García‐Ulloa, M. I. , A. E. Escalante , A. Moreno‐Letelier , L. E. Eguiarte , and V. Souza . 2021. “Evolutionary Rescue of an Environmental *Pseudomonas otitidis* in Response to Anthropogenic Perturbation.” Frontiers in Microbiology 11: 2020. 10.3389/fmicb.2020.563885 PMC7856823 33552002

[ece374364-bib-0031] Garnier‐Géré, P. , and L. Chikhi . 2013. “Population Subdivision, Hardy–Weinberg Equilibrium and the Wahlund Effect.” Encyclopedia of Life Sciences.

[ece374364-bib-0032] Garvie, L. 1998. “Population Genetic Structure and Conservation Status of an Endemic Habitat‐Specialist Macroinvertebrate ( *Cherax robustus* ) in South‐East Queensland. Honours thesis, Queensland University of Technology.”

[ece374364-bib-0033] Goudet, J. 2005. “Hierfstat, a Package for r to Compute and Test Hierarchical F‐Statistics.” Molecular Ecology Notes 5, no. 1: 184–186.

[ece374364-bib-0034] Gruber, B. , P. J. Unmack , O. F. Berry , and A. Georges . 2018. “Dartr: An r Package to Facilitate Analysis of SNP Data Generated From Reduced Representation Genome Sequencing.” Molecular Ecology Resources 18, no. 3: 691–699. 10.1111/1755-0998.12745 29266847

[ece374364-bib-0035] Haig, S. M. , E. A. Beever , S. M. Chambers , et al. 2006. “Taxonomic Considerations in Listing Subspecies Under the U.S. Endangered Species Act.” Conservation Biology 20, no. 6: 1584–1594. 10.1111/j.1523-1739.2006.00530.x 17181793

[ece374364-bib-0036] Hanson, J. M. , K. J. Welsh , P. T. Moss , and P. Gadd . 2023. “Implications of Sea Level Variability on the Formation and Evolution of Subtropical Rainbow Beach Patterned Fen Complexes, Queensland, Australia.” Holocene 33, no. 1: 49–60.

[ece374364-bib-0037] Hewitt, G. 2000. “The Genetic Legacy of the Quaternary Ice Ages.” Nature 405, no. 6789: 907–913. 10.1038/35016000 10879524

[ece374364-bib-0039] Hughes, J. M. , J. A. Huey , and D. J. Schmidt . 2013. “Is Realised Connectivity Among Populations of Aquatic Fauna Predictable From Potential Connectivity?” Freshwater Biology 58, no. 5: 951–966.

[ece374364-bib-0038] Hughes, J. , M. Ponniah , D. Hurwood , S. Chenoweth , and A. Arthington . 1999. “Strong Genetic Structuring in a Habitat Specialist, the Oxleyan Pygmy Perch *Nannoperca oxleyana* .” Heredity 83, no. 1: 5–14. 10.1038/sj.hdy.6885390 10447698

[ece374364-bib-0040] Josić, D. , E. Çoraman , I. Waurick , et al. 2024. “Cryptic Hybridization Between the Ancient Lineages of Natterer's Bat ( *Myotis nattereri* ).” Molecular Ecology 33, no. 13: e17411. 10.1111/mec.17411 38785347

[ece374364-bib-0041] Kilian, A. , P. Wenzl , E. Huttner , et al. 2012. “Diversity Arrays Technology: A Generic Genome Profiling Technology on Open Platforms.” In Data Production and Analysis in Population Genomics: Methods and Protocols, 67–89. Data Production and Analysis in Population Genomics, Methods and Protocols. 10.1007/978-1-61779-870-2_5 22665276

[ece374364-bib-0042] Knight, J. T. , A. H. Arthington , G. S. Holder , and R. B. Talbot . 2012. “Conservation Biology and Management of the Endangered Oxleyan Pygmy Perch *Nannoperca oxleyana* in Australia.” Endangered Species Research 17, no. 2: 169–178.

[ece374364-bib-0043] Leach, L. 2011. “Hydrology and Physical Setting of North Stradbroke Island.” Proceedings of the Royal Society of Queensland 117: 21–46.

[ece374364-bib-0044] Leonard, S. , and J. Nott . 2016. “A Holocene Fluvial Archive of the Mulgrave River, Notheastern Australia: Influence of Tropical Cyclones and Sediment Delivery to the Great Barrier Reef.” Palaeogeography, Palaeoclimatology, Palaeoecology 441: 845–853.

[ece374364-bib-0045] Lewis, S. E. , C. R. Sloss , C. V. Murray‐Wallace , C. D. Woodroffe , and S. G. Smithers . 2013. “Post‐Glacial Sea‐Level Changes Around the Australian Margin: A Review.” Quaternary Science Reviews 74: 115–138.

[ece374364-bib-0046] Lovrenčić, L. , M. Temunović , L. Bonassin , F. Grandjean , C. Austin , and I. Maguire . 2022. “Climate Change Threatens Unique Genetic Diversity Within the Balkan Biodiversity Hotspot–the Case of the Endangered Stone Crayfish.” Global Ecology and Conservation 39: e02301.

[ece374364-bib-0047] Lowe, K. , J. G. Castley , and J.‐M. Hero . 2013. “Acid Frogs Can Stand the Heat: Amphibian Resilience to Wildfire in Coastal Wetlands of Eastern Australia.” International Journal of Wildland Fire 22, no. 7: 947–958.

[ece374364-bib-0049] Marshall, J. C. , P. M. Negus , A. L. Steward , and G. B. McGregor . 2011. “Distributions of the Freshwater Fish and Aquatic Macroinvertebrates of North Stradbroke Island Are Differentially Influenced by Landscape History, Marine Connectivity and Habitat Preference.” Proceedings of the Royal Society of Queensland 117: 239–260.

[ece374364-bib-0050] McGuire, K. 1968. “Land Development for Beef Production in the Wallum.” Quarterly Review of the Rural Economy 21, no. 3: 140.

[ece374364-bib-0051] Meyer, E. 2006. “National Recovery Plan for the Wallum Sedgefrog and Other Wallum‐Dependent Frog Species, Queensland. Parks and Wildlife Service”.

[ece374364-bib-0052] Mijangos, J. L. , B. Gruber , O. Berry , C. Pacioni , and A. Georges . 2022. “dartR v2: An Accessible Genetic Analysis Platform for Conservation, Ecology and Agriculture.” Methods in Ecology and Evolution 13, no. 10: 2150–2158.

[ece374364-bib-0053] Mijangos, J. L. , C. Pacioni , P. B. S. Spencer , and M. D. Craig . 2015. “Contribution of Genetics to Ecological Restoration.” Molecular Ecology 24, no. 1: 22–37. 10.1111/mec.12995 25377524

[ece374364-bib-0054] Morgan, D. , and S. Beatty . 2005. Fish and crayfish fauna of Ellen Brook, Cowaramup Brook and Gunyulgup Brook in the Cape to Cape Region of Western Australia.

[ece374364-bib-0055] Moritz, C. 1994. “Defining ‘Evolutionarily Significant Units’ for Conservation.” Trends in Ecology & Evolution 9, no. 10: 373–375. 10.1016/0169-5347(94)90057-4 21236896

[ece374364-bib-0056] Moss, P. , J. Tibby , F. Shapland , et al. 2015. “Patterned Fen Formation and Development From the Great Sandy Region, South‐East Queensland, Australia.” Marine and Freshwater Research 67, no. 6: 816–827.

[ece374364-bib-0057] Nichols, H. J. 2017. “The Causes and Consequences of Inbreeding Avoidance and Tolerance in Cooperatively Breeding Vertebrates.” Journal of Zoology 303, no. 1: 1–14.

[ece374364-bib-0058] Nunziata, S. O. , and D. W. Weisrock . 2018. “Estimation of Contemporary Effective Population Size and Population Declines Using RAD Sequence Data.” Heredity 120, no. 3: 196–207. 10.1038/s41437-017-0037-y PMC5836589 29269932

[ece374364-bib-0059] Oliveira, E. A. , M. F. Perez , L. A. C. Bertollo , et al. 2020. “Historical Demography and Climate Driven Distributional Changes in a Widespread Neotropical Freshwater Species With High Economic Importance.” Ecography 43, no. 9: 1291–1304.

[ece374364-bib-0060] Olsen, K. C. , W. H. Ryan , A. A. Winn , et al. 2020. “Inbreeding Shapes the Evolution of Marine Invertebrates.” Evolution 74, no. 5: 871–882. 10.1111/evo.13951 PMC7383701 32191349

[ece374364-bib-0061] Page, T. J. 2021. Nomination to Change the Conservation Class of *Cherax robustus* Under the Queensland Nature Conservation Act 1992. Department of Environment and Science.

[ece374364-bib-0062] Page, T. J. , and J. M. Hughes . 2007a. “Phylogeographic Structure in an Australian Freshwater Shrimp Largely Pre‐Dates the Geological Origins of Its Landscape.” Heredity 98, no. 4: 222–231. 10.1038/sj.hdy.6800932 17213864

[ece374364-bib-0063] Page, T. J. , and J. M. Hughes . 2007b. “Radically Different Scales of Phylogeographic Structuring Within Cryptic Species of Freshwater Shrimp (Atyidae: Caridina).” Limnology and Oceanography 52, no. 3: 1055–1066.

[ece374364-bib-0064] Page, T. J. , J. C. Marshall , and J. M. Hughes . 2012. “The World in a Grain of Sand: Evolutionarily Relevant, Small‐Scale Freshwater Bioregions on Subtropical Dune Islands.” Freshwater Biology 57, no. 3: 612–627.

[ece374364-bib-0065] Page, T. J. , S. Sharma , and J. M. Hughes . 2004. “Deep Phylogenetic Structure Has Conservation Implications for Ornate Rainbowfish (Melanotaeniidae: *Rhadinocentrus ornatus* ) in Queensland, Eastern Australia.” Marine and Freshwater Research 55, no. 2: 165–172.

[ece374364-bib-0066] Paradis, E. 2010. “Pegas: An R Package for Population Genetics With an Integrated–Modular Approach.” Bioinformatics 26, no. 3: 419–420. 10.1093/bioinformatics/btp696 20080509

[ece374364-bib-0067] Paradis, E. , and K. Schliep . 2018. “Ape 5.0: An Environment for Modern Phylogenetics and Evolutionary Analyses in R.” Bioinformatics 35, no. 3: 526–528. 10.1093/bioinformatics/bty633 30016406

[ece374364-bib-0068] Passos, T. U. , J. M. Webster , J. C. Braga , et al. 2019. “Paleoshorelines and Lowstand Sedimentation on Subtropical Shelves: A Case Study From the Fraser Shelf, Australia.” Australian Journal of Earth Sciences 66, no. 4: 547–565.

[ece374364-bib-0070] Payenberg, T. H. , R. Boyd , J. Beaudoin , et al. 2006. The Filling of An Incised Valley By Shelf Dunes—An Example From Hervey Bay, East Coast of Australia.

[ece374364-bib-0071] Pearl, H. , T. Ryan , M. Howard , Y. Shimizu , and A. Shapcott . 2022. “DNA Barcoding to Enhance Conservation of Sunshine Coast Heathlands.” Diversity 14, no. 6: 436.

[ece374364-bib-0073] Pearl, H. , T. Ryan , M. Howard , Y. Shimizu , and A. Shapcott . 2024. “Conserving Diversity, Distinctiveness and Connectivity in the Sunshine Coast Heathlands, Queensland, Australia.” Austral Ecology 49, no. 3: e13504.

[ece374364-bib-0074] Phillimore, A. B. , and I. P. Owens . 2006. “Are Subspecies Useful in Evolutionary and Conservation Biology?” Proceedings of the Royal Society B: Biological Sciences 273, no. 1590: 1049–1053. 10.1098/rspb.2005.3425 PMC1560251 16600880

[ece374364-bib-0075] Reitzel, A. M. , S. Herrera , M. J. Layden , M. Q. Martindale , and T. M. Shank . 2013. “Going Where Traditional Markers Have Not Gone Before: Utility of and Promise for RAD Sequencing in Marine Invertebrate Phylogeography and Population Genomics.” Molecular Ecology 22, no. 11: 2953–2970. 10.1111/mec.12228 PMC3669247 23473066

[ece374364-bib-0076] Renwick, J. 2006. “Population Structure and Genetic Diversity of Southeast Queensland Populations of the Wallum Froglet, *Crinia tinnula* (Tschudi). PhD Thesis. Queensland University of Technology.”

[ece374364-bib-0077] Saitou, N. , and M. Nei . 1987. “The Neighbor‐Joining Method: A New Method for Reconstructing Phylogenetic Trees.” Molecular Biology and Evolution 4, no. 4: 406–425. 10.1093/oxfordjournals.molbev.a040454 3447015

[ece374364-bib-0078] Sattler, P. S. 1999. The Conservation Status of Queensland's Bioregional Ecosystems. Environmental Protection Agency, Queensland Government.

[ece374364-bib-0091] Scully, O. 2024. “Dietary Studies of the Acid‐Specialist Crayfish *Cherax Robustus*.” Undergraduate Thesis, University of the Sunshine Coast.

[ece374364-bib-0079] Sharma, S. , and J. M. Hughes . 2009. “Genetic Structure and Phylogeography of Freshwater Shrimps (*Macrobrachium australiense* and *Macrobrachium tolmerum* ): The Role of Contemporary and Historical Events.” Marine and Freshwater Research 60, no. 6: 541–553.

[ece374364-bib-0080] Sharma, S. , and J. M. Hughes . 2011. “Genetic Structure and Phylogeography of Two Freshwater Fishes, *Rhadinocentrus ornatus* and *Hypseleotris compressa* , in Southern Queensland, Australia, Inferred From Allozymes and Mitochondrial DNA.” Journal of Fish Biology 78, no. 1: 57–77. 10.1111/j.1095-8649.2010.02843.x 21235546

[ece374364-bib-0081] Shuker, J. D. , C. A. Simpkins , and J.‐M. Hero . 2016. “Determining Environmental Limits of Threatened Species: The Example of the Wallum Sedgefrog *Litoria olongburensis* .” Ecosphere 7, no. 6: e01384.

[ece374364-bib-0082] Shulmeister, J. , T. Rittenour , N. Patton , et al. 2024. “Chronology and Evolution of the World's Largest Sand Island: K'gari (Fraser Island), South East Queensland, Australia.” Quaternary Science Reviews 328: 108529.

[ece374364-bib-0083] Smith, G. , S. Glendinning , and T. Ventura . 2023. “Transcriptomic Changes Following Induced De‐Masculinisation of Australian Red Claw Crayfish *Cherax quadricarinatus* .” International Journal of Molecular Sciences 24, no. 4: 3292. 10.3390/ijms24043292 PMC9966960 36834703

[ece374364-bib-0084] Tibby, J. , C. Barr , J. C. Marshall , et al. 2017. “Persistence of Wetlands on North Stradbroke Island (South‐East Queensland, Australia) During the Last Glacial Cycle: Implications for Quaternary Science and Biogeography.” Journal of Quaternary Science 32, no. 6: 770–781.

[ece374364-bib-0085] UNEP . 2022. “Global Peatlands Assessment: The State of the World's Peatlands ‐ Evidence for Action Toward the Conservation, Restoration, and Sustainable Management of Peatlands.”

[ece374364-bib-0086] Wang, Q. , J. Hu , T. Wu , et al. 2025. “Population Patterns and Dynamics of *Ilisha elongata* (Clupeiformes: Pristigasteridae) Revealed by Target Enrichment Data.” Evolutionary Applications 18, no. 8: e70142. 10.1111/eva.70142 PMC12329003 40778091

[ece374364-bib-0087] Waters, J. M. , C. P. Burridge , D. Craw , and J. S. Albert . 2026. “Geological Processes Shaping Freshwater Biodiversity: A Synthesis of Global Evidence.” Biological Reviews 101, no. 3: 1568–1581. 10.1002/brv.70135 PMC13149781 41631706

[ece374364-bib-0088] Weir, B. S. , and C. C. C. ockerham . 1984. “Estimating F‐Statistics for the Analysis of Population Structure.” Evolution 38, no. 6: 1358–1370. 10.1111/j.1558-5646.1984.tb05657.x 28563791

[ece374364-bib-0090] Woolschot, L. , J. M. Hughes , and S. E. Bunn . 1999. “Dispersal Among Populations of *Caridina* sp.(Decapoda: Atyidae) in Coastal Lowland Streams, South‐Eastern Queensland, Australia.” Marine and Freshwater Research 50, no. 7: 681–688.

[ece374364-bib-0089] Wright, S. 1965. “The Interpretation of Population Structure by F‐Statistics With Special Regard to Systems of Mating.” Evolution 19: 395–420.

